# In Vivo Engineered CAR-T Cell Therapy: Lessons Built from COVID-19 mRNA Vaccines

**DOI:** 10.3390/ijms26073119

**Published:** 2025-03-28

**Authors:** Sikun Meng, Tomoaki Hara, Yutaka Miura, Yasuko Arao, Yoshiko Saito, Kana Inoue, Takaaki Hirotsu, Andrea Vecchione, Taroh Satoh, Hideshi Ishii

**Affiliations:** 1 Department of Medical Data Science, Center of Medical Innovation and Translational Research, Osaka University Graduate School of Medicine, Yamadaoka 2-2, Suita, Osaka 565-0871, Japan; 2 Laboratory for Chemistry and Life Science, Institute of Integrated Research, Institute of Science Tokyo, 4259 Nagatsutacho, Midori-ku, Yokohama 226-8501, Japan; 3 Hirotsu Bio Science Inc., Chiyoda-Ku, Tokyo 102-0094, Japan; 4 Department of Clinical and Molecular Medicine, University of Rome “Sapienza”, Santo Andrea Hospital, Via di Grottarossa, 1035, 00189 Rome, Italy; 5 Center for Cancer Genomics and Precision Medicine, Osaka University Hospital, Yamadaoka 2-2, Suita, Osaka 565-0871, Japan

**Keywords:** CAR-T therapy, mRNA vaccines, mRNA technology, lipid nanoparticles, immune response regulation

## Abstract

Chimeric antigen receptor T cell (CAR-T) therapy has revolutionized cancer immunotherapy but continues to face significant challenges that limit its broader application, such as antigen targeting, the tumor microenvironment, and cell persistence, especially in solid tumors. Meanwhile, the global implementation of mRNA vaccines during the COVID-19 pandemic has highlighted the transformative potential of mRNA and lipid nanoparticle (LNP) technologies. These innovations, characterized by their swift development timelines, precise antigen design, and efficient delivery mechanisms, provide a promising framework to address some limitations of CAR-T therapy. Recent advancements, including mRNA-based CAR engineering and optimized LNP delivery, have demonstrated the capacity to enhance CAR-T efficacy, particularly in the context of solid tumors. This review explores how mRNA-LNP technology can drive the development of in vivo engineered CAR-T therapies to address current limitations and discusses future directions, including advancements in mRNA design, LNP optimization, and strategies for improving in vivo CAR-T functionality and safety. By bridging these technological insights, CAR-T therapy may evolve into a versatile and accessible treatment paradigm across diverse oncological landscapes.

## 1. Introduction

Chimeric antigen receptor T cell (CAR-T) therapy has achieved remarkable success in the treatment of hematologic malignancies. However, it still faces significant challenges, including relapse and antigen escape, limited persistence and long-term efficacy, and high manufacturing costs [1,2]. Perhaps most frustratingly, despite its promising potential, CAR-T therapy has shown only limited progress in the treatment of solid tumors, with outcomes remaining suboptimal to date [3]. Addressing these challenges requires innovative solutions to optimize efficacy and expand the reach of CAR-T therapy.

The rapid emergence and global spread of COVID-19 at the end of 2019 catalyzed the development of diverse vaccine technologies, with mRNA and lipid nanoparticle (LNP) platforms emerging as the most significant breakthroughs. These technologies have demonstrated unparalleled advantages, including accelerated development timelines, precise antigen design, and flexible delivery mechanisms [4]. The implementation of mRNA vaccines, exemplified by Pfizer-BioNTech (BNT162b2) and Moderna (mRNA-1273), highlights the game-changing capabilities of these innovations to address long-standing hurdles in cancer immunotherapy.

By leveraging insights from mRNA vaccine development, CAR-T therapy may unlock new pathways for optimization, especially in the treatment of solid tumors. This review explores the application prospects of these cutting-edge technologies in CAR-T cell therapy, highlighting their potential to address current challenges and enhance therapeutic outcomes.

## 2. Current Status and Challenges of CAR-T Cell Therapy

### 2.1. Hematological Malignancies

In the treatment of hematological malignancies, CAR-T therapies targeting the CD19 antigen have demonstrated remarkable efficacy. Products such as Kymriah and Yescarta have been approved and widely applied for managing acute lymphoblastic leukemia (ALL) and diffuse large B-cell lymphoma [5,6]. Kymriah achieved an overall response rate of 81% and a complete remission rate (CR rate) of 60% in ALL pediatric patients, offering a critical treatment option for patients with refractory hematological cancers [7]. However, despite these encouraging initial outcomes, long-term follow-up studies have shown a decline in CR rates [8], revealing the persistent challenges CAR-T therapies face in treating blood cancers.

Antigen escape is a major obstacle to the success of CAR-T therapies in hematological malignancies [9]. Some tumor cells evade immune recognition by downregulating or completely losing target antigens, such as CD19 [10], or through the expansion of pre-existing antigen-negative clones, such as CD19^−^ subpopulations that persist from baseline, ultimately leading to disease relapse [11]. Additionally, the limited persistence of CAR-T cells in vivo significantly affects their therapeutic efficacy. CAR-T cell persistence is limited by the progressive loss of functionality, which is primarily driven by chronic antigen stimulation, metabolic dysregulation, and sustained inhibitory receptor signaling. This often leads to their inability to self-renew and perform effective immune responses over time [12]. Resistance to repeated treatments is another critical issue, as some patients exhibit diminished responses to subsequent CAR-T therapy re-infusion, further constraining the long-term effectiveness of these treatments [13].

Side effects remain a significant barrier to the broader application of CAR-T therapy. The most common and potentially severe complications include cytokine release syndrome (CRS) and immune effector cell-associated neurotoxicity syndrome (ICANS), both of which are closely linked to the proliferation of CAR-T cells and the patient’s immune status [10]. Additionally, the high cost of treatment and the complexity of the manufacturing process further limit the accessibility of CAR-T therapy. These challenges stem from labor-intensive procedures, such as leukapheresis, genetic modification, and the ex vivo expansion of CAR-T cells, which substantially increase both time and economic burdens [14].

### 2.2. Solid Tumors

While the success of CAR-T therapy in blood cancers provides a promising foundation for its application in solid tumors, the more complex obstacles posed by its distinct biological characteristics greatly diminish its efficacy. Major challenges include the lack of suitable antigens, the highly immunosuppressive tumor microenvironment (TME), and the limited persistence of CAR-T cells in solid tumors [15]. Overcoming these barriers requires further research and innovative technological advancements to enhance the effectiveness of CAR-T therapy in the context of solid tumors.

The lack of suitable antigens can be interpreted in two dimensions. First, there is a scarcity of truly tumor-specific antigens. Many common antigens, such as HER2, are also expressed in normal tissues, posing the risk of “on-target/off-tumor” (OTOT) toxicity, as observed in cases where anti-HER2 CAR-T cells cause fatal respiratory distress or severe gastrointestinal hemorrhage [16,17]. On the other hand, antigen heterogeneity within solid tumors further complicates treatment. Different tumor cells within the same tumor can express diverse antigens, making it difficult for CAR-T cells to target a single antigen to eradicate the entire tumor [18]. This heterogeneity enables antigen-negative tumor cells to evade immune recognition, ultimately leading to relapse and treatment resistance [19].

The TME creates multiple barriers that hinder the efficacy of CAR-T cell therapy in solid tumors. The dense stromal structure surrounding solid tumors, composed of collagen fibers and fibroblasts, restricts CAR-T cell infiltration, while hypoxic and low-pH conditions further impair CAR-T cell activity and persistence [20,21]. Additionally, the TME is enriched with immunosuppressive components, including cytokines such as TGF-β and IL-10, as well as inhibitory immune cells like regulatory T cells (Tregs) and myeloid-derived suppressor cells, all of which suppress CAR-T activation and function [22,23]. Notably, the interaction between tumors and the immune system often categorizes solid tumors into “hot” (immune-infiltrated) or “cold” (immune-desert) environments. “Cold” tumors, characterized by a lack of immune cell infiltration, deficiency of chemokines, and a stroma-dense structure, can prevent the effective recruitment of cytotoxic T cells, including CAR-T cells [24].

## 3. Critical Elements Behind the Rapid Development of mRNA Vaccines

The mRNA vaccine represents a groundbreaking innovation, utilizing LNPs to deliver mRNA into cells, where it is directly translated into antigenic proteins within the cytoplasm, mimicking aspects of natural viral infection. Through the presentation of antigens via MHC I molecules, CD8^+^ T cells are activated, while MHC II molecules stimulate CD4^+^ T cells, which in turn assist B cells in producing antibodies [25]. This unique mechanism enables mRNA vaccines to simultaneously elicit robust T-cell responses and antibody production. The ability of mRNA vaccines to induce highly efficient immune responses is closely linked to their distinctive molecular and biological features, alongside advantages such as rapid development, flexible administration, and reduced side effects, making them a powerful tool in vaccine development. However, such a sophisticated design is the result of decades of scientific effort. This section briefly reviews the development of its core technologies and highlights the key factors underlying the implementation of COVID-19 mRNA vaccines.

### 3.1. Breakthroughs in mRNA Technology

**Figure 1:**
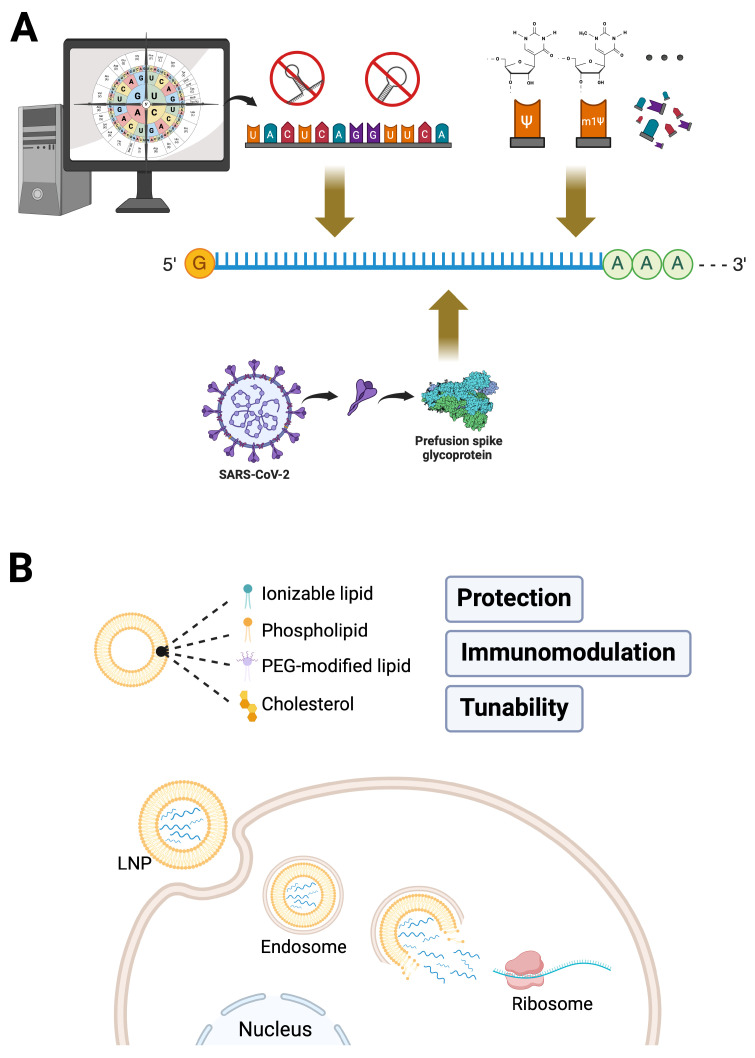
mRNA-LNP Platforms: The Foundation of COVID-19 mRNA Vaccines. (**A**) Engineering of mRNA, exemplified by COVID-19 vaccines encoding the pre-fusion-stabilized spike glycoprotein, demonstrates key strategies, including codon optimization, avoidance of secondary structures, and nucleotide modifications (e.g., pseudouridine, N1-methylpseudouridine), that enhance mRNA stability, translational efficiency, and minimize immunogenicity. (**B**) Lipid nanoparticles (LNPs), composed of ionizable lipids, phospholipids, cholesterol, and PEG-modified lipids, provide protection, immune modulation, and tunable delivery properties for effective mRNA transport. LNPs facilitate efficient cytoplasmic delivery of mRNA via pH-sensitive endosomal escape, a critical mechanism applicable both to vaccines and emerging in vivo CAR-T cell therapies.

The development of mRNA technology has been a long and challenging journey, marked by several critical stages. In the 1960s, mRNA was identified as the key intermediary between DNA and protein synthesis [26,27], and scientists had successfully translated mRNA in vitro [28]. In the late 1980s, Malone and colleagues pioneered the use of synthetic cationic lipids to deliver mRNA, demonstrating its ability to express proteins in animal cells [29]. Around the turn of the century, Katalin Karikó and Drew Weissman discovered that synthetic mRNA was recognized by Toll-like receptors as a threat, triggering extensive inflammatory responses [30]. They overcame this by rearranging the chemical bonds of uridine to create pseudouridine, which significantly reduced immunogenicity [31]. In the 21st century, the advent of LNP delivery systems resolved major hurdles in mRNA delivery and stability, paving the way for its broader application in vaccines and therapeutics. By the 2010s, mRNA technology had entered clinical research, with companies such as Moderna and BioNTech spearing its use in infectious diseases and cancer immunotherapy. The COVID-19 pandemic in 2020 served as a proving ground for this technology, with the rapid development and deployment of mRNA vaccines, such as BNT162b2 and mRNA-1273, showing its ability to respond swiftly and effectively to global health crises [32,33]. The remarkable implementation of COVID-19 vaccines has vividly demonstrated the transformative potential of mRNA as a highly adaptable and precisely controllable molecule, allowing extensive modifications to suit diverse needs and offering unprecedented flexibility in vaccine and therapeutic development (Figure 1A).

#### 3.1.1. Precise Antigen Design

mRNA vaccines enable the precise encoding of target antigens. The spike protein (S protein) of SARS-CoV-2 is a trimeric structure with a highly dynamic receptor-binding domain (RBD). In the RBD-up conformation, the RBD is exposed and capable of binding to the ACE2 receptor on host cells, playing a critical role in viral entry [34]. The natural state of the S protein prior to host cell fusion, known as the prefusion conformation, is essential for initiating membrane fusion and, thus, represents a key step in viral infection. Consequently, interventions targeting this conformation can effectively block viral entry. Studies have shown that antibodies targeting the prefusion conformation of the S protein, such as RBD-specific antibodies, exhibit stronger neutralizing activity, as they prevent the virus from interacting with the ACE2 receptor. By introducing artificial modifications—such as beneficial proline substitutions and stabilizing mutations—the prefusion conformation can be stabilized, preventing its transition to the postfusion state [35,36]. This stabilization allows vaccines to more effectively mimic natural infection, thereby eliciting robust immune responses.

#### 3.1.2. Optimization of mRNA Sequences

The optimization of mRNA sequences is a critical step in improving vaccine efficacy. Thoughtful design of mRNA sequences significantly enhances their stability, translational efficiency, and antigen expression. This section focuses on the pivotal strategies of codon optimization, minimizing secondary structure formation, and the optimization of 5′ and 3′ untranslated regions (UTRs) to improve mRNA performance.

Codons are the fundamental coding units of mRNA, and their usage frequency varies across host cells, a phenomenon known as codon bias. By replacing rare codons in foreign genes with codons preferred by the host cell, researchers can enhance ribosomal recognition and increase translation efficiency [37]. Interestingly, to address the rapid evolution of SARS-CoV-2, codon deoptimization (CDO) has been employed in the development of the live-attenuated vaccine candidate CDO-7N-1. This approach involves substituting frequently used codons in the viral genome with rarely used ones in the host cell, significantly reducing viral replication capacity and attenuating virulence while preserving broad immunogenicity against multiple variants [38].

mRNA, as a single-stranded nucleic acid, often forms secondary structures such as hairpins and stem loops through base pairing, which can hinder ribosome binding and movement along the mRNA strand. To mitigate this issue, researchers have employed computational algorithms to optimize mRNA sequences by predicting and minimizing regions prone to forming stable secondary structures. By strategically rearranging nucleotide sequences to create a more open configuration, ribosomal accessibility and translation efficiency are significantly improved, thereby boosting the production of antigenic proteins [39].

The 5′ and 3′ UTRs of mRNA play pivotal roles in regulating mRNA stability, translation efficiency, and half-life [40]. The 5′ UTR is particularly important for enhancing ribosome binding efficiency [41]. Recently, polysome profiling and deep learning were used to analyze a library of 280,000 randomized 5′ UTR sequences, resulting in a predictive model and the design of novel 5′ UTRs that precisely modulate ribosome loading levels to optimize protein expression [42]. On the other hand, the 3′ UTR is integral to controlling mRNA stability and translation efficiency [43]. Furthermore, the poly(A) tail has become a widely utilized and essential component of mRNA technology, although its relationship with translation and mRNA decay still requires further refinement and understanding [44]. Emerging studies have examined various aspects of the poly(A) tail, such as its length and dynamic behavior in specific biological processes, uncovering its diverse roles in natural mRNA translation and regulation [45,46]. The findings above provide valuable insights for designing synthetic mRNA to enhance its stability and functionality.

#### 3.1.3. Nucleoside Modifications

To reduce the immune response triggered by cellular recognition of mRNA as foreign RNA, modified nucleosides are commonly incorporated into mRNA [47]. These modifications enhance stability and expression levels while minimizing side effects. Pseudouridine (Ψ) is one of the most frequently used modifications, effectively reducing recognition by the innate immune system through Toll-like receptors (TLR7 and TLR8), thereby improving translation efficiency [31]. Furthermore, research has shown that N1-methylpseudouridine (m1Ψ) enhances these effects even further and has been widely employed in the BNT162b2 and mRNA-1273 vaccines [48,49]. In addition, some companies, such as CureVac, have opted for sequence engineering strategies to prevent mRNA immune activation rather than relying on nucleoside modifications [50], indicating that nucleoside modification and sequence optimization represent two complementary approaches for reducing immunogenicity and improving mRNA efficacy.

### 3.2. Advances in Delivery Systems

Delivery systems form a versatile foundation for the practical application of mRNA technologies, which play indispensable roles in protecting mRNA, enhancing delivery efficiency, and achieving precise targeting. The evolution of lipid-based delivery systems dates back to the 1960s with the introduction of liposomes. These vesicles, composed of phospholipid bilayers, could encapsulate both hydrophilic and lipophilic drugs, marking the beginning of a new era in drug delivery [51,52]. However, limitations in stability and drug-loading capacity prompted the development of solid lipid nanoparticles in the 1990s, which utilized solid lipids to achieve greater physical stability [53]. In the 2000s, nanostructured lipid carriers were introduced, combining solid and liquid lipids to improve drug-loading efficiency and release control, particularly for cancer therapies and sustained drug release [54]. The 2010s witnessed groundbreaking advances with the emergence of ionizable LNPs, which leverage pH-responsive properties to enable efficient nucleic acid delivery [55]. This technology achieved notable recognition in 2018 with the FDA approval of Patisiran, the first LNP-based nucleic acid therapy [56]. Finally, in the 2020s, LNP technology reached its pinnacle with the delivery of COVID-19 mRNA vaccines, where it played a crucial role in protecting mRNA and enhancing intracellular delivery, as demonstrated by the Pfizer-BioNTech and Moderna vaccines [57].

LNPs are typically composed of four fundamental lipid components: ionizable lipids, phospholipids, cholesterol, and polyethylene glycol (PEG)-modified lipids [58]. Ionizable lipids, which are neutral at physiological pH, become positively charged in acidic environments such as endosomes. This pH-sensitive property facilitates the fusion of LNPs with the endosomal membrane, enabling the release of mRNA into the cytoplasm. Phospholipids contribute to the bilayer structure of LNPs, mimicking the lipid bilayer of cell membranes, thereby enhancing interactions with membrane structures and improving delivery efficiency. Cholesterol plays a critical role in stabilizing the LNP structure and increasing membrane fluidity, allowing easier fusion with the cell membrane and maintaining stability within the body. On the other hand, PEG-modified lipids extend the circulation time of LNPs by preventing rapid clearance by the immune system. Additionally, PEG reduces the aggregation of LNPs in the bloodstream, further improving delivery efficiency [58,59]. This functional integration of lipid components makes LNPs highly effective carriers for mRNA delivery. Next, we discuss its pivotal role in mRNA vaccines (Figure 1B).

#### 3.2.1. Protecting mRNA Integrity and Ensuring Efficient Delivery

mRNA is inherently a highly degradable molecule, rendering it extremely unstable in physiological environments and necessitating effective delivery systems for its protection [60]. LNPs achieve this by encapsulating mRNA within their phospholipid bilayer structure and cholesterol components, providing stability and preventing degradation before reaching target cells. The efficient delivery capability of LNPs stems from their ionizable lipid content, which exhibits unique pH-responsive properties. At the neutral pH of the bloodstream (~7.4), ionizable lipids remain uncharged, ensuring LNP stability. Upon entering cells through endocytosis, LNPs are enclosed within endosomes, where the acidic environment (pH ~5.0–6.0) protonates these lipids, resulting in a positive charge. This charge alteration disrupts the negatively charged endosomal membrane, enabling LNPs to escape and release mRNA into the cytoplasm [61]. This pH-sensitive characteristic grants LNPs dual functionality: maintaining stability in systemic circulation and enabling precise cargo release within target cells, enhancing efficacy while minimizing side effects.

#### 3.2.2. Balancing Immunogenicity: Relatively Low Risk with Enhanced Efficacy

Compared to viral vectors, LNPs exhibit relatively low immunogenicity, mitigating the risk of triggering strong immune responses, which makes them safer choices for mRNA delivery [62]. Moreover, LNPs have the unique potential to act as adjuvants, modestly stimulating innate immune responses to increase vaccine efficacy [63,64]. Studies have shown that ionizable lipids in LNPs drive adjuvant activity by inducing the production of cytokines and chemokines such as IL-1β, IL-6, and GM-CSF. These molecules activate innate immune pathways and support robust adaptive immune responses [63,65,66]. Meanwhile, PEG-modified lipids prolong circulation time by reducing immune clearance; however, they may elicit mild immune reactions, particularly in individuals with pre-existing antibodies against PEG, potentially leading to hypersensitivity or reduced delivery efficiency [67,68]. Optimizing the concentration and structure of PEG-modified lipids is, therefore, critical to balancing stability and immunogenicity.

#### 3.2.3. Tunability and Industrial Scalability

LNPs offer remarkable tunability, allowing precise targeting through modifications, such as specific ligands or component adjustments, and enabling the delivery of nucleic acids to specific tissues or cell types [69]. This adaptability makes LNPs a versatile platform for various therapeutic applications, including mRNA vaccines, gene therapies, and siRNA-based treatments.

In addition to tunability, LNPs exhibit industrial scalability, largely enabled by the ethanol-loading technique. This method temporarily alters lipid configurations in an ethanol environment to encapsulate nucleic acids, with LNPs reassembling into stable nanoparticles upon ethanol removal [70]. The technique is both efficient and reproducible, making it ideal for large-scale manufacturing and supporting the rapid production of LNP-based therapies, including mRNA vaccines and siRNA treatments such as Patisiran.

## 4. From Vaccines to CAR-T Therapy: Bridging Challenges with Technological Insights

**Figure 2:**
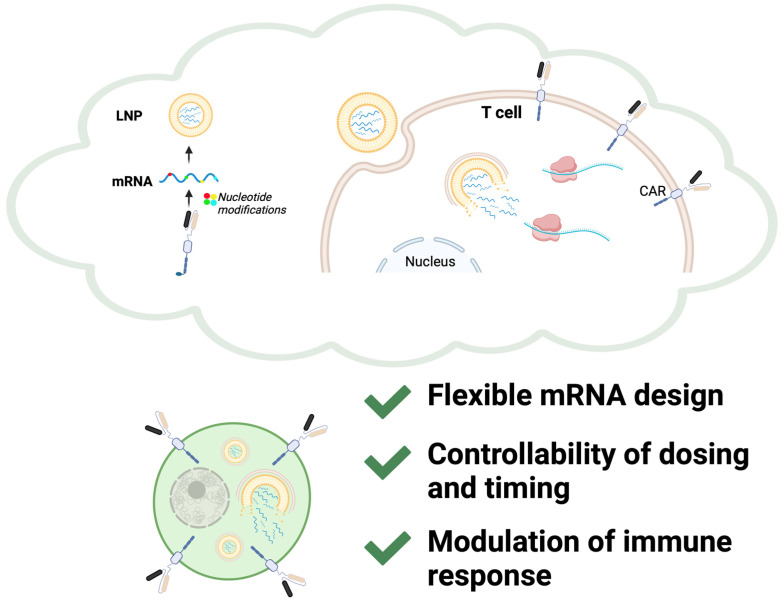
Schematic Representation of mRNA-Based CAR-T Cell Engineering and Key Advantages. The schematic illustrates mRNA-based engineering of CAR-T cells using LNP technology. In this process, CAR-encoding mRNA, incorporating optimized nucleotide modifications, is encapsulated into LNPs for targeted in vivo delivery. Upon cellular uptake, mRNA undergoes efficient cytoplasmic translation into CAR proteins, transiently expressed on T cell surfaces, to mediate precise tumor targeting. Key advantages of this mRNA-LNP platform include flexible and personalized mRNA design, precise control over dosing and timing (enabling pulsed administration strategies), and modulation of immune responses to optimize therapeutic efficacy while minimizing potential adverse effects. These features collectively position mRNA-LNP-induced CAR-T therapy as a promising approach for adaptable, safe, and efficient cancer immunotherapy.

Current CAR-T cell therapy primarily relies on ex vivo manufacturing, accompanied by the limitations discussed above. In vivo CAR-T engineering has emerged as a promising alternative to traditional ex vivo manufacturing, leveraging either viral or non-viral delivery approaches [71]. Viral vectors, such as adeno-associated viruses and lentiviruses, have been explored for direct CAR gene delivery; however, concerns over insertional mutagenesis, prolonged CAR expression, and manufacturing complexity have driven interest toward non-viral strategies [72]. The remarkable advantages demonstrated by mRNA vaccines—such as rapid production, precise antigen design, and the ability to modulate immune responses—raise an intriguing question: might these attributes hold the key to addressing the myriad challenges faced by CAR-T cell therapy, from optimizing efficacy to enhancing durability and ensuring safety? Parayath et al. pioneered an injectable nanocarrier system capable of delivering in vitro-transcribed mRNA encoding CAR or TCR directly to circulating T cells, effectively reprogramming them in vivo to combat diseases such as leukemia and prostate cancer [73]. Building on this platform, Rurik et al. utilized LNPs to transiently generate CAR-T cells targeting cardiac fibroblasts, successfully reducing fibrosis and restoring cardiac function in murine models of heart injury [74]. These studies underscore the transformative potential of mRNA and LNP technologies in overcoming traditional CAR-T therapy limitations (Figure 2). Table 1 summarizes the representative studies that highlight advancements in mRNA-LNP delivery strategies for in vivo CAR-T therapy.

### 4.1. Perspectives from mRNA Technology

mRNA technology allows for the precise design of multi-antigen sequences, enabling solutions to the antigen heterogeneity commonly observed in solid tumors. By incorporating multiple tumor antigens, CAR-T cells can be engineered to target diverse antigenic profiles within the whole tumor, reducing the likelihood of immune escape and improving therapeutic efficacy. Additionally, this flexibility allows for rapid adaptation to newly emerging antigens in cases of tumor recurrence and metastasis. Liu et al. explored a sequential CAR-T therapy in murine pancreatic cancer models, which first targets fibroblast activation protein-positive cancer-associated fibroblasts to remodel the TME and subsequently affects CLDN18.2-positive tumor cells. This strategy demonstrated superior efficacy compared with the simultaneous infusion of both CAR-T cell types [78]. If implemented with mRNA-based CAR-T cells, such sequential therapies could be realized with substantially reduced time and economic costs, leveraging the rapid production and adaptability of mRNA technology while maintaining therapeutic efficacy. Borrowing from the versatility of mRNA vaccines, multi-target CAR-T cells could represent a promising strategy to overcome the challenges of tumor antigen heterogeneity.

The transient nature of mRNA offers significant advantages in terms of safety and flexibility [74]. Unlike viral vectors that induce permanent CAR expression, mRNA-based CAR-T cells allow for controlled, short-term expression, thereby reducing the risk of severe adverse effects such as OTOT toxicity, CRS and ICANS. This transient expression also provides the opportunity to fine tune therapeutic timing and dosing, enabling precise modulation of CAR-T cell activity in response to patient-specific needs and minimizing the likelihood of prolonged or excessive immune activation. This flexibility positions mRNA-based CAR-T cells as safer and more adaptable alternatives in the evolving landscape of cancer immunotherapy.

### 4.2. Perspectives from LNP Delivery Systems

Next-generation CAR-T cell therapy fundamentally relies on innovations in the delivery of genetic information, making delivery systems indispensable components for optimizing transduction efficiency and targeting specificity. The precise delivery and intracellular release capabilities of LNP technology offer a promising avenue to enhance the genetic modification efficiency of CAR-T cells, thereby advancing their therapeutic potential. Recent studies have evidenced the feasibility of using ionizable LNPs for mRNA-based CAR-T cell engineering, achieving levels of CAR expression comparable to those of traditional electroporation methods while significantly reducing cytotoxicity [75,79].

Furthermore, LNP technology provides an innovative pathway to enhance the antitumor efficacy of CAR-T cell therapy through immune response modulation [80]. A recent study optimized LNP formulations for mRNA cancer vaccines, enabling intravenous delivery to antigen-presenting cells (APCs), like dendritic cells and macrophages, in the spleen. This stimulated the maturation of APCs and the secretion of cytokines, including type I interferons and IP-10, which promoted tumor-specific CD8^+^ T cell infiltration, suppressed tumor growth and prolonged survival in preclinical models [81]. By acting as adjuvants, LNPs can be designed to fine-tune the innate immune activation associated with CAR-T therapy, potentially amplifying antitumor immune responses while improving CAR-T cell persistence within the TME.

## 5. Future Directions

CAR-T cell therapy has already achieved considerable clinical success and continues to evolve as a cornerstone of cancer immunotherapy. Building on the transformative advancements of COVID-19 mRNA vaccines, as analyzed earlier, mRNA-based CAR-T therapy has compelling advantages, including cost efficiency, shorter development timelines, and enhanced design flexibility. These attributes position mRNA as a highly promising platform for the next generation of CAR-T therapies. Nevertheless, to drive CAR-T cell therapy toward comprehensive breakthroughs, several critical areas of optimization must be addressed. This section focuses on three key directions: refining nucleotide modifications in mRNA to enhance therapeutic performance, advancing LNP delivery systems for greater specificity and scalability, and harnessing controlled immune activation to overcome the immunosuppressive TME. These strategies collectively aim to unlock the full potential of mRNA-based CAR-T cell therapy.

### 5.1. Refining Nucleotide Modifications in mRNA Technology

Nucleotide modifications are essential for improving the stability and translational efficiency of mRNA, as well as for fine-tuning the immune response to exogenous RNA. While these modifications extend mRNA expression and sustain therapeutic effects, they can also help reduce mRNA immunogenicity to counterbalance the increased likelihood of excessive immune activation due to its prolonged presence. In addition to pseudouridine (Ψ), many other nucleotide modifications are deeply involved in various stages of disease progression and have emerged as promising therapeutic targets. Harnessing their potential for therapeutic mRNA modification to ensure both efficacy and safety in clinical applications remains a compelling area of exploration.

#### 5.1.1. Methylation-Based Modifications

Building on the structure of pseudouridine, N1-methylpseudouridine (m1Ψ) offers enhanced immune evasion capabilities by reducing recognition by toll-like receptors. In a study on multiple sclerosis, m1Ψ-modified mRNA was used to encode disease-associated self-antigens. By delivering antigens non-inflammatorily and promoting Treg expansion, this m1Ψ-modified mRNA effectively suppressed disease progression in several mouse models of experimental autoimmune encephalomyelitis [82]. m1Ψ has been regarded as a key advancement in mRNA therapeutics, offering improved stability, reduced immunogenicity, and enhanced translational efficiency for diverse applications.

N^6^-Methyladenosine (m6A) is one of the most prevalent internal modifications of mRNA and plays a critical role in regulating mRNA stability, translational efficiency, and nuclear export. Mechanically, this modification interacts with certain proteins, including ‘writers’ such as the METTL3-METTL14 complex, which catalyzes its addition, ‘readers’ such as the YTHDF family, which bind to m6A-modified RNA to mediate downstream effects, and ‘erasers’ such as FTO and ALKBH5, which remove the methyl group, maintaining the dynamic regulation of RNA metabolism [83,84]. m6A, which is naturally abundant in endogenous mRNA, has emerged as a promising target for addressing multiple complex diseases, including cancer [85], and has also been explored for its ability to increase the efficacy of mRNA vaccines and therapeutic mRNA applications. In our previous work, we introduced m6A modifications to the therapeutic mRNA, leading to further acceleration of the anti-tumor response, convincingly demonstrating the potential of m6A-equipped mRNA to optimize therapeutic efficacy.

5-Methylcytosine (m5C) is a well-characterized modification in DNA, essential for gene regulation and stability. Although it is present at low levels in eukaryotic mRNA, recent studies have begun to reveal its biological significance [86]. Enzymes like NSUN2 catalyze m5C modifications, protecting RNA from cleavage under stress [86]. Notably, m5C at specific mitochondrial tRNA sites, such as C34 in tRNAMet, enhances oxidative phosphorylation, a process with critical implications in tumor metabolism, by improving mitochondrial translation efficiency [87]. We might actively leverage these functions in therapeutic mRNA design, where they could enhance stability and translation efficiency, thereby boosting the performance of mRNA-based treatments.

In addition, other modifications, such as 5-methyluridine (m5U) and N1-methyladenosine (m1A), have drawn significant attention. m5U has been shown to participate in ribosomal function by regulating tRNA transport and stability. It also enhances mRNA translation efficiency and improves its stability [88]. Studies suggest that alterations in m5U expression levels under certain pathological conditions may interact with disease processes, making it a potential therapeutic target or an optimization strategy for mRNA modifications [88]. m1A, on the other hand, is notable for its role in regulating RNA secondary structure and protein-RNA interactions. Specifically, the methylation characteristic of m1A alters the base-pairing capacity of adenosine, inducing a bend in the RNA strand at the modification site. This structural change can expose or obscure protein-binding sites on RNA, influencing its interactions with other molecules. In cancer-related research, m1A demethylation has been closely linked to metabolic reprogramming and tumor progression, further highlighting the importance of m1A regulation in disease processes [89].

#### 5.1.2. Non-Methylation-Based Modifications

2′-O-Methylation, despite its name, refers to the modification of the 2′ hydroxyl group of the ribose sugar rather than the nucleotide base itself. It is a common and significant RNA modification found in tRNA, rRNA, and mRNA, and plays a critical role in enhancing RNA stability and reducing immunogenicity. In tRNA, 2′-O-methylguanosine (Gm18) has been shown to effectively suppress the activation of TLR7 and TLR8, thereby attenuating innate immune responses [90]. If the 2′-O-methyl group is absent from Gm18, its immunosuppressive effect is substantially diminished. However, when combined with m5U to form 2′-O-methylthymidine (Tm or m5Um), the RNA demonstrates markedly enhanced immune evasion from TLR recognition [91]. This dual modification not only effectively suppresses immune activation but also improves RNA stability and translational efficiency.

2-Thiouridine (s2U) is a common tRNA modification that enhances tRNA stability and functionality through the sulfur modification of the uridine base. Studies have shown that a low level of s2U modification is associated with translational stalling at lysine (LysAAA) codons. In combination with 5-carboxymethylaminomethyl (mcm5) modification, s2U forms mcm5s2U, which is widely present at wobble positions in tRNAs for lysine, glutamate, and glutamine, and plays a critical role in translational regulation. Under stress conditions, such as artemisinin resistance in the malaria parasite Plasmodium falciparum, reduced levels of mcm5s2U modification alter codon usage preferences, prioritizing the translation of specific proteins, thereby enabling the parasite to adapt to drug pressure through targeted protein expression strategies [92]. Intriguingly, s2U and related modifications hold promise for optimizing translation efficiency and selectivity in therapeutic applications.

In summary, epigenetic modifications of mRNA play a crucial role in regulating various physiological and pathological processes. From tumor metabolism and drug resistance to the precise modulation of tRNA functionality, many fascinating discoveries have emerged from these fields of study. These findings, in turn, offer valuable insights and foundational knowledge for advancing therapeutic mRNA modifications. In other words, this domain remains a largely untapped frontier, presenting immense opportunities for further exploration and innovation. Nevertheless, we should also take into account that recent studies have suggested that certain modifications, such as m1Ψ, may induce +1 ribosomal frameshifting, potentially impacting translational fidelity, although no human-derived reports have indicated adverse effects [93]. Future research could focus on several key areas: optimizing combinations of multiple modifications, such as investigating the synergistic effects of m6A with other modifications like m1Ψ, to achieve an optimal balance between immunogenicity and antitumor activity; developing cancer-specific modification strategies tailored to different tumor types, enhancing therapeutic efficacy while minimizing side effects; and delving deeper into the mechanisms of these modifications, leveraging single-cell technologies and multi-omics analyses to uncover the systemic impacts of mRNA modifications on immune regulation, thereby providing a solid theoretical foundation for next-generation CAR-T therapies.

### 5.2. Optimizing Targeting Strategies in LNP Systems

The synthesis of LNPs is highly controllable, offering significant potential for tailoring solutions to specific challenges. Targeted delivery via LNPs has become a key focus in drug delivery research. By fine tuning their structure, surface modifications, and other properties, scientists can enhance the specificity of LNPs for particular cells or tissues, thereby minimizing off-target effects and reducing side effects. Several widely used approaches are available for optimizing the targeting capabilities of all types of LNPs, as discussed below.

#### 5.2.1. Passive Targeting and Physicochemical Properties

Passive targeting primarily relies on the interactions between specific microenvironments and the physicochemical properties of nanocarriers. For example, in tumor or inflamed tissues, newly formed blood vessels often have incomplete structures with enlarged endothelial gaps, allowing macromolecular substances like nanoparticles to pass through more easily and accumulate within the tumor microenvironment. Additionally, the lack of effective lymphatic drainage in tumor tissues hinders the clearance of nanoparticles, prolonging their retention time. This phenomenon, known as the enhanced permeability and retention effect, facilitates the accumulation of LNPs in tumor tissues while minimizing their impact on healthy tissues [94]. Beyond size and shape control, the composition of LNP lipids plays a critical role in enabling passive targeting. For example, the Spleen Selective ORgan Targeted strategy incorporates anionic lipids such as 18:1 PA into LNP formulations to mitigate their typical liver accumulation and achieve targeted delivery to extrahepatic tissues [76]. This modification operates via charge-based targeting by lowering the pKa of LNPs, enhancing their compatibility with the mildly acidic microenvironment of the spleen and altering their organ distribution. Similarly, by customizing lipid tail length (shorter tails are preferred), bond types (ester bonds outperform amide bonds), and head group structures (methyl groups show superior performance), LNP delivery efficiency to lymph nodes can be significantly improved [95]. Moreover, a partial substitution of cholesterol with hydroxycholesterol in LNP formulations alters the binding kinetics with Niemann–Pick C1 enzymes, reducing endosomal recycling and enhancing T cell delivery by 1.8 to 2 fold [96]. The critical role of lipid composition in shaping LNP performance has also driven advancements in screening and development methodologies. Li, B. and colleagues utilized combinatorial chemistry and machine learning to establish a high-throughput platform for ionizable lipid screening, enabling the prediction of lipid performance while identifying optimal lipid combinations [97]. These innovative techniques, which transitioned incremental improvements into transformative advancements, significantly accelerate the development of LNPs and expand the boundaries of the field.

#### 5.2.2. Active Targeting via Surface Modifications

Active targeting enhances the specificity of LNPs for target cells or tissues by modifying their surface with specific molecules such as antibodies, ligands, or small molecules. The antibody-conjugated LNP platform developed by Billingsley, M. M., is a notable example, which achieved precise T-cell targeting by incorporating antibodies against markers like CD3, CD5, and CD7 on the LNP surface. This approach significantly reduced off-target distribution to non-target tissues, such as the liver [98]. Similarly, a study on LNP-mediated mRNA delivery for treating hereditary retinal disorders employed M13 phage display technology to identify retinal-targeting peptides for LNP modification. This enabled specific recognition of photoreceptor cells within the highly barriered retinal structure, demonstrating excellent biocompatibility and markedly reduced off-target toxicity [99].

#### 5.2.3. Stimuli-Responsive Targeting

Another strategy involves designing LNPs to respond to external stimuli. LNPs made from sugar alcohol fatty acid monoesters exhibit lower or upper critical solution temperature behavior, allowing them to undergo structural changes at specific temperatures. This property is particularly advantageous in pathological environments with temperature variations, such as tumor sites with localized heating, enabling controlled drug release for more precise delivery [100]. In another study, Fe_3_O_4_ superparamagnetic nanoparticles were incorporated into LNPs, leveraging external magnetic fields to drive mechanical targeting and accumulation of LNPs in tumor regions. These LNPs were designed as dual-contrast nanoparticles with both optical absorption and magnetic properties. By integrating photoacoustic and magneto-ultrasound imaging technologies, these LNPs demonstrated high sensitivity for tumor imaging, offering significant potential in precision diagnostics and targeted therapies [101].

Enhancing the blood–brain barrier (BBB)penetration is a promising approach for improving the efficacy of certain brain tumor treatments and in vivo CAR-T strategies. Techniques such as focused ultrasound and microbubble-assisted BBB opening have been investigated for transiently increasing BBB permeability, allowing LNP-mediated mRNA delivery into brain tissues [102]. Peptide-functionalized LNPs, including those modified with T7, RVG29, AP2, and mApoE peptides, have further improved brain-specific mRNA transfection and may offer potential applications for future in vivo CAR-T therapies [103,104]. Modern LNP technologies integrate biological engineering, nanomaterial design, and external modulation strategies to enhance the specificity and efficacy of drug delivery while minimizing side effects. In the future, LNP targeting strategies are likely to become increasingly refined and personalized, and tailored to meet the therapeutic demands of various diseases.

### 5.3. Balancing Immune Response

In the context of immune response regulation, COVID-19 vaccines and cancer immunotherapy have fundamentally different requirements. COVID-19 vaccines aim to ensure that the immune system recognizes and remembers the pathogen while minimizing type I interferon responses and excessive immune activation. This approach is crucial for avoiding systemic inflammation and immune-related side effects. Conversely, in cancer therapy, particularly for overcoming the immunosuppressive “cold” tumor microenvironment, a robust inflammatory response is indispensable. Such activation serves as a critical mechanism to counteract immune suppression and stimulate effective antitumor immunity [105].

In the previous sections, we have thoroughly discussed how mRNA modifications and LNP system design influence immune responses. In cancer therapy, these strategies must be optimized to meet the unique demands of immune activation. Unlike vaccines for infectious diseases, cancer immunotherapies often prefer intravenous administration to rapidly and robustly activate the immune system, overcoming the immunosuppressive tumor microenvironment [106]. Additionally, the application of combination therapies has shown significant promise. For example, in patients who are unresponsive to PD-1 antibody therapies, mRNA cancer vaccines targeting specific tumor-associated antigens have significantly reactivated the immune system by triggering type I interferon responses via TLR pathways, thereby reversing tumor resistance [107].

Beyond these approaches, an important strategy to sustain therapeutic effects while minimizing excessive immune activation is intermittent administration (pulsed dosing). Instead of a single high-dose injection, repeated low-dose mRNA administrations can maintain CAR-T cell functionality over an extended period while reducing the risk of prolonged immune activation. This approach, validated in mRNA vaccine and gene therapy studies, provides a controlled mechanism to fine-tune therapeutic response and avoid potential immune-related toxicities. By integrating optimized mRNA modifications with strategic dosing regimens, CAR-T therapy can achieve a sustained therapeutic window while mitigating the risks associated with excessive immune stimulation.

Ultimately, the key to effective cancer treatment lies in fully mobilizing and harnessing the patient’s immune system, maximizing its anti-tumor potential through precise design. Future research should focus on developing strategies for the dual regulation of immune responses: enhancing inflammatory reactions to activate anti-tumor immunity, especially in the context of immune-excluded “cold tumors”, while simultaneously maintaining tight control over immune response intensity to avoid “overactivation,” which could lead to CRS or other immune-related adverse events. Achieving an “on-demand” immune response that balances efficacy and safety will be crucial for advancing cancer immunotherapy.

## 6. Conclusions

Lessons learned from the development of COVID-19 mRNA vaccines, particularly breakthroughs in mRNA engineering and lipid nanoparticle delivery systems, offer transformative strategies to address the current limitations of CAR-T therapy. By leveraging transient gene expression, improved delivery mechanisms, and controlled immune responses, mRNA-based CAR-T therapies have the potential to overcome existing barriers and significantly enhance therapeutic efficacy. Future advancements in mRNA design, LNP formulations, and targeted delivery strategies are expected to further optimize in vivo CAR-T therapies, improving their persistence, safety, and cost. Additionally, integrating cutting-edge innovations such as artificial intelligence-driven CAR optimization, nanomaterial-based delivery enhancements, and immune microenvironment modulation will continue to expand the applicability of mRNA technologies in CAR-T cell therapy. These advancements collectively highlight the critical role of mRNA-based approaches in shaping the next generation of cancer immunotherapy.

## Figures and Tables

**Table 1:** Representative studies showcasing key advancements in mRNA-LNP delivery for in vivo CAR-T therapy.

Study/Company	mRNA-LNP System	Key Findings	Reference
Billingsley et al., 2020	Ionizable LNPs (C14-4 LNPs) optimized for T cell transfection	First study demonstrating LNP-based CAR mRNA delivery. Screened 24 different LNPs, identifying C14-4 as the optimal formulation for efficient mRNA transfection into T cells.	[75]
Rurik et al., 2022	CD5-targeted LNPs delivering nucleoside-modified mRNA encoding FAP CAR	CD5-targeted LNPs selectively transfected T cells and generated transient CAR-T cells. Successfully reduced fibrosis and restored cardiac function in a mouse model of heart failure.	[74]
Álvarez-Benedicto et al., 2023	Spleen SORT LNPs for antibody-free T cell targeting	By optimizing LNP composition, the organ tropism was shifted from primarily targeting the liver to selectively transfecting T cells in the spleen. Achieved efficient transfection of CD3^+^ T cells without requiring antibody modifications. Successfully generated in vivo CAR-T cells, leading to prolonged survival in a B-cell lymphoma mouse model and reduced liver off-target effects.	[76]
Hamilton et al., 2023	mRNA/siRNA Co-Delivery LNPs for CAR expression and PD-1 knockdown	Engineered co-delivery LNPs capable of simultaneously delivering CAR mRNA and siRNA to knock down PD-1 in T cells. Demonstrated enhanced CAR-T cell efficacy while minimizing systemic PD-1 inhibition, reducing potential immune-related side effects.	[77]
Capstan Therapeutics	CD8-targeted LNPs delivering sequence-enhanced mRNA encoding anti-CD19 CAR	Developed CPTX2309, a novel T cell-selective mRNA-LNP platform (CellSeeker tLNP). Achieved rapid and preferential CD8^+^ T cell transfection, generating functional CAR-T cells without lymphodepletion. Demonstrated tumor clearance and B cell depletion in preclinical models.	
Orna Therapeutics	Circular RNA (oRNA)-LNPs with optimized internal ribosome entry sites (IRESs) for enhanced CAR expression	Developed in situ CAR (isCAR™) therapy, using circular RNA (oRNA) instead of linear mRNA for greater stability. Immunotropic LNPs preferentially delivered oRNA to T cells and other immune cells. Achieved dose-dependent tumor regression in a humanized mouse model of leukemia.	

## Abbreviations

CAR-T	Chimeric antigen receptor T-cell
LNP	Lipid nanoparticle
ALL	Acute lymphoblastic leukemia
CR rate	complete remission rate
CRS	Cytokine release syndrome
ICANS	Immune effector cell-associated neurotoxicity syndrome
TME	Tumor microenvironment
OTOT	On-target/off-tumor (toxicity)
Tregs	Regulatory T cells
RBD	Receptor-binding domain
UTRs	Untranslated regions
CDO	Codon deoptimization
TLR	Toll-like receptor
PEG	Polyethylene glycol
APCs	Antigen-presenting cells
m1Ψ	N1-methylpseudouridine
m6A	N^6^-Methyladenosine
m5C	5-Methylcytosine
m5U	5-methyluridine
m1A	N1-methyladenosine
s2U	2-Thiouridine
mcm5	5-carboxymethylaminomethyl
